# Unsupervised Fault Diagnosis of a Gear Transmission Chain Using a Deep Belief Network

**DOI:** 10.3390/s17071564

**Published:** 2017-07-04

**Authors:** Jun He, Shixi Yang, Chunbiao Gan

**Affiliations:** 1 The State Key Laboratory of Fluid Power and Mechatronic Systems, College of Mechanical Engineering, Zhejiang University, Hangzhou 310027, China; hjshenhua@zju.edu.cn (J.H.); cb_gan@zju.edu.cn (C.G.); 2 The Key Laboratory of Advanced Manufacturing Technology of Zhejiang Province, College of Mechanical Engineering, Zhejiang University, Hangzhou 310027, China

**Keywords:** deep belief networks, unsupervised feature learning, fault diagnosis, gear transmission chain

## Abstract

Artificial intelligence (AI) techniques, which can effectively analyze massive amounts of fault data and automatically provide accurate diagnosis results, have been widely applied to fault diagnosis of rotating machinery. Conventional AI methods are applied using features selected by a human operator, which are manually extracted based on diagnostic techniques and field expertise. However, developing robust features for each diagnostic purpose is often labour-intensive and time-consuming, and the features extracted for one specific task may be unsuitable for others. In this paper, a novel AI method based on a deep belief network (DBN) is proposed for the unsupervised fault diagnosis of a gear transmission chain, and the genetic algorithm is used to optimize the structural parameters of the network. Compared to the conventional AI methods, the proposed method can adaptively exploit robust features related to the faults by unsupervised feature learning, thus requires less prior knowledge about signal processing techniques and diagnostic expertise. Besides, it is more powerful at modelling complex structured data. The effectiveness of the proposed method is validated using datasets from rolling bearings and gearbox. To show the superiority of the proposed method, its performance is compared with two well-known classifiers, i.e., back propagation neural network (BPNN) and support vector machine (SVM). The fault classification accuracies are 99.26% for rolling bearings and 100% for gearbox when using the proposed method, which are much higher than that of the other two methods.

## 1. Introduction

Abnormal operations induced by gear or bearing failures should be detected as early as possible to avoid serious and even fatal accidents. A variety of methods have been applied for the diagnosis of rotating machinery based on vibration and acoustic signals [1,2], thermal features [3] and oil debris [4], among which the vibration based analysis is one of the most commonly used technique [5,6]. According to the characteristics of the data, it is feasible to detect abnormalities in vibration signals and make decisions about the health conditions of gear or bearing by employing appropriate data analysis algorithms such as empirical mode decomposition (EMD) [7], spectral kurtosis [8], wavelet analysis [9], and time synchronous averaging [10]. Nevertheless, most of these methods depend on careful observation and recognition of the corresponding features of the vibration signals to identify the faults, which require a great deal of expertise to apply them successfully. On the other hand, because of the complexity of the equipment and the variety of the fault categories, massive real-time data are required to fully inspect the health conditions of the equipment. It would be labour-intensive and time-consuming for diagnosticians to analyze massive data based on appropriate methods. Therefore, simpler approaches are needed which allow relatively unskilled operators to make reliable and rapid decisions with less expertise and labour. Artificial intelligence (AI) techniques, which can effectively analyze massive amounts of fault data and automatically provide accurate diagnosis results, have been successfully applied to detect abnormalities in rotating machinery [11,12,13,14,15]. Li et al. proposed a novel feature extraction and selection scheme to obtain a more compact feature subset, and then applied four types of AI techniques for the hybrid fault diagnosis of a gearbox [1]. Cheng et al. proposed a fault diagnosis framework for rolling bearing based on scale invariant feature transform, kernel principal component analysis and SVM [16]. Samanta et al. extracted time-domain features and employed ANNs and SVM to diagnose bearing faults [17]. Zhao et al. presented a new back propagation neural network (BPNN) based on an improved shuffled frog leaping algorithm for the fault diagnosis of bearings [18].

Throughout the previous researches, we find that ANNs are one of the most commonly used classifiers in intelligent fault diagnosis, among which back propagation neural network (BPNN) is the representative one based on supervised learning [19]. It consists of the input layer, the output layer and the hidden layers, and is generally trained to find a function that can best map a set of inputs and the corresponding outputs by using back propagation algorithm. The SVM is another well-known classifier that is based on statistical learning theory, VC dimension and structural risk minimization. This method has better generalization than ANNs have and can solve the learning problem of smaller number of samples quite well. Meanwhile, it has advantages on nonlinearity problems and high dimensional pattern recognition problems [20,21,22]. Nevertheless, one of the salient challenges to these techniques is the capability to capture relevant health condition information from the massive datasets associated with practical applications. Modern rotating machinery components produce datasets that are usually complex and noisy because of actual operating conditions. Accurate modelling of such complex data can hardly be achieved by conventional AI methods because they can accommodate only a small number of non-linear operations [23,24,25,26]. To overcome this deficiency, domain specific features that are more relevant to the health conditions are preferred for subsequent fault classification. Nevertheless, developing domain specific features through feature extraction and feature selection is overwhelmingly dependent on prior knowledge about signal processing techniques and diagnostic expertise, which is unpractical in industrial applications because of the significant variety of equipment and working conditions [23]. These problems have greatly limited the practical application of conventional AI diagnosis methods. 

Deep learning [26,27,28,29] has recently proven its capability for unsupervised feature learning in various fields such as speech recognition [30,31], motion capture [32], visual recognition [33,34], and physiology [35,36]. This new technique takes full advantage of unsupervised feature learning to extract features from unlabelled time domain data instead of features selected by a human operator, eliminating the conventional dependence on prior knowledge of signal processing techniques and diagnostic expertise. Besides, the deep architectures in the networks are more capable of modelling complex structures in the data compared with conventional shallow methods. Therefore, deep learning represents a substantial improvement over conventional AI techniques and offers the potential to address the challenges facing fault diagnosis of rotating machinery [37,38,39]. Li et al., applied a Gaussian-Bernoulli deep Boltzmann machine (GDBM) to diagnose fault patterns in gearbox and bearing [39]. Tran et al., presented a new method based on a deep belief network (DBN) for the fault diagnosis of valves in reciprocating compressors [40]. Gao et al., utilized the deep quantum inspired neural network (DQINN) to the aircraft fuel system fault diagnosis [41]. Jeong et al., developed autonomous orbit shape recognition systems for the purpose of rotor diagnosis using the deep learning algorithm [42]. However, there still exist manual signal processing or feature selection techniques in these methods, and the powerful ability of deep learning in unsupervised feature learning has not been fully investigated. Jia et al., integrated a Fourier transform (FFT) and deep neural networks (DNNs) to diagnose faults in rotating machinery [43]. In this method, the frequency spectra of the measured signals calculated by the FFT were used as input data for the DNNs. Nevertheless, the deviations caused by the assumptions of linearity, periodicity and stationarity could not be avoided when the Fourier spectral analysis is applied to nonstationary data [44]. Therefore, it is favourable to train the deep network directly from the raw signals in the time domain.

This paper proposes a novel AI method based on a DBN to achieve unsupervised feature learning and automatic fault diagnosis of a gear transmission chain. In this method, the DBN is first pre-trained layer by layer in an unsupervised manner and then fine-tuned with a back propagation (BP) algorithm under supervision. The unsupervised process aims to obtain representative features characterizing the health conditions of machinery from the raw data directly, and the supervised process is implemented to discover the discriminative information from these features. Besides, the genetic algorithm is used to optimize the structural parameters of the network. Compared to the conventional AI methods, the proposed method can adaptively exploit robust features related to the faults from the unlabelled time domain data, which little field expertise is needed. Besides, the proposed deep network has superiorities to model complex structured data, thus can discover the discriminative information of these data and achieve accurate classification. To illustrate the application of the proposed method, the rest of this paper is organized as follows: Section 2 introduces this method, in which the DBN is trained directly by the unlabelled time domain data to learn domain specific features and a back propagation (BP) algorithm is used to fine-tune the network to achieve fault classification. Section 3 describes the problems that limit supervised learning methods applied to the fault diagnosis of rotating machinery. Section 4 presents the diagnosis results of experimental rolling bearing data and public gearbox fault datasets based on the proposed method. Section 5 discusses these results, and Section 6 summarizes the conclusions.

## 2. Methods 

In this section, a DBN-based AI method is proposed to achieve unsupervised feature learning and automatic classification. 

### 2.1. DBN Conceptual Framework

**Figure 1:**
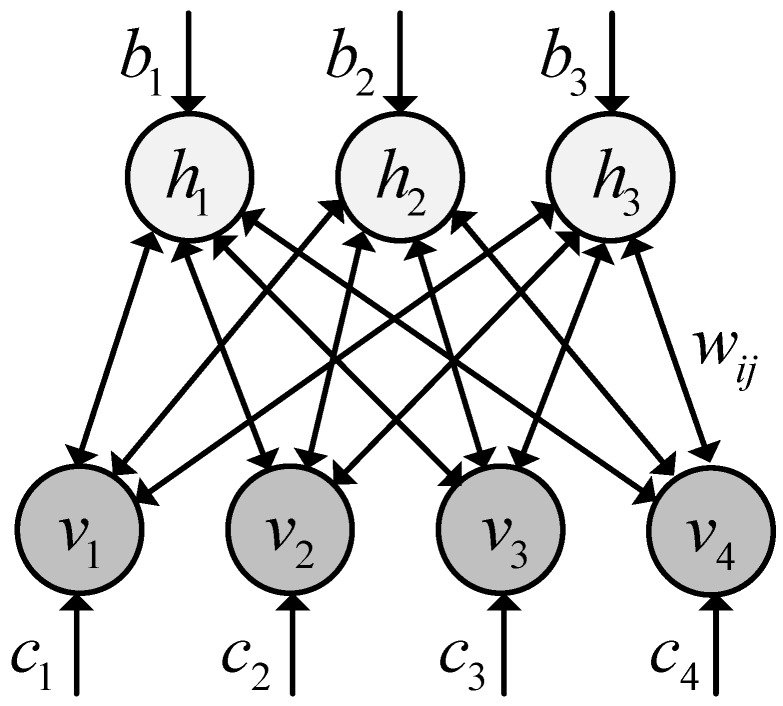
Restricted Boltzmann machine (RBM) architecture.

DBN is an unsupervised feature learning model with deep architecture. Each layer of the DBN is a restricted Boltzmann machine (RBM) [28,35,45], which is a generative probabilistic model with input units (visible units) v, and hidden units h. The visible and hidden units are connected with a weight matrix W and have bias vectors c and b, respectively. There are no visible-visible connections and no hidden-hidden connections. The architecture of the RBM is depicted in Figure 1.

For given visible and hidden units, the energy function is defined as:

(1) $$E\left(v,h\right)=-\overset{V}{\underset{i=1}{\sum }}\overset{H}{\underset{j=1}{\sum }}w_{ij}v_{i}h_{j}-\overset{V}{\underset{i=1}{\sum }}c_{i}v_{i}-\overset{H}{\underset{j=1}{\sum }}b_{j}h_{j}$$

where $v_{i}$ and $h_{j}$ are the binary states of visible unit i and hidden unit j; $c_{i}$ and $b_{j}$ are their biases and $w_{ij}$ is the weight between them; V and H are the number of visible and hidden units. The joint distribution for the visible and hidden units is defined via the energy function as:

(2) $$P\left(v,h\right)=\frac{1}{Z}e^{-E\left(v,h\right)}$$

where Z is the partition function that ensures that the distribution is normalized. For binary visible and hidden units, the probability that hidden unit $h_{j}$ is activated given visible vector v, and the probability that visible unit $v_{i}$ is activated given hidden vector h, are given by:

(3) $$P\left(h_{j}|v\right)=\delta \left(b_{j}+\underset{i}{\sum }w_{ij}v_{i}\right)$$

(4) $$P\left(v_{i}|h\right)=\delta \left(c_{i}+\underset{j}{\sum }w_{ij}h_{j}\right)$$

respectively, where δ(⋅) is the activation function. The logistic function $\delta \left(x\right)=1/\left(1+e^{-x}\right)$ is a common choice for the activation function. Equation (3) describes the positive phase learning process that transforms the input data from a high-dimensional space into characteristic vectors in a low-dimensional space, and Equation (4) describes the negative phase learning process that reconstructs the input data from the characteristic vectors. The parameter W, c and b, are trained simultaneously to minimize the reconstruction error. The mean square error (MSE):

(5) $$J_{\mathrm{MSE}}=\frac{1}{M}\overset{M}{\underset{m=1}{\sum }}\left(∥x^{m}-z^{m}{∥}^{2}\right)$$

is usually used as the standard loss function, where $x^{m}$ is an input sample from a dataset ${\left\{x^{m}\right\}}_{m=1}^{M}$ and $z^{m}$ is the corresponding reconstruction.

**Figure 2:**
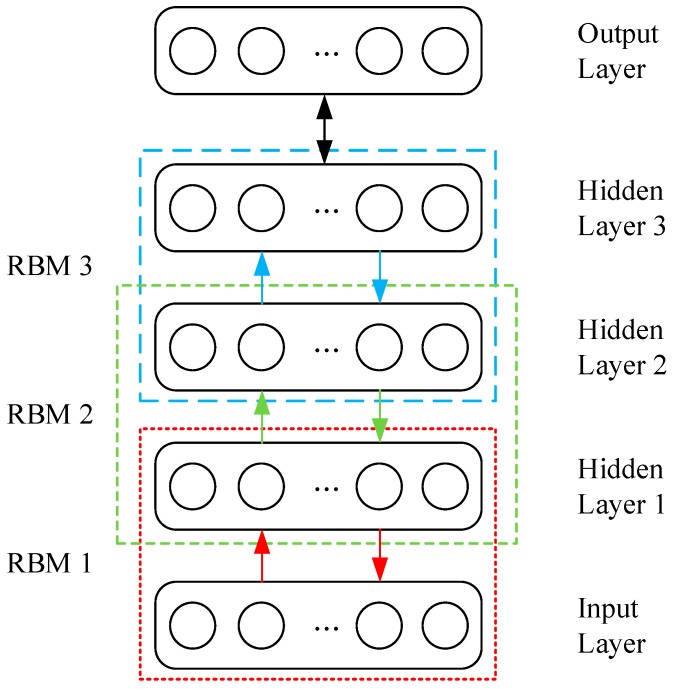
A DBN structure with three stacked RBMs [37].

Several RBMs can be stacked to produce a DBN, the output of a lower-layer RBM is the input to a higher-layer RBM. Figure 2 displays a DBN structure with three stacked RBMs, layer 1 (input layer) and layer 2 (hidden layer 1) form the first RBM, layer 2 (hidden layer 1) and layer 3 (hidden layer 2) form the second RBM, layer 3 (hidden layer 2) and layer 4 (hidden layer 3) form the third RBM, and finally is the output layer. It should be noted that the hidden layer of the first RBM, i.e., layer 2, is also the visible layer of the second RBM, and so is the second RBM and the third RBM.

### 2.2. Training Process of DBN Classifier Model

The DBN classifier model is trained according to two main steps: (1) pre-training of each individual RBM layer by layer with unsupervised learning and (2) further fine-tuning of the DBN with a back propagation (BP) algorithm for classification.

In the first step, each RBM can be trained by performing a stochastic gradient descent on the negative log-likelihood probability of the training data. The gradient of the negative logarithmic probability of the visible layer with respect to parameter W is defined as:

(6) $$\frac{\partial \log P\left(v\right)}{\partial w_{ij}}=\langle v_{i}h_{j}\rangle_{data}-\langle v_{i}h_{j}\rangle_{model}$$

where ${\langle \cdot \rangle }_{data}$ denotes the expectation under the distribution of the data, and ${\langle \cdot \rangle }_{model}$ ndenotes the expectation under the distribution of the model. In practice, because the exact computation of ${\langle v_{i}h_{j}\rangle }_{model}$ is exceedingly difficult, an approximation referred to as contrastive divergence after *k* iterations of Gibbs sampling (often *k* = 1) is usually used to train the RBM [46,47]. When given input data $x^{m}$ from a dataset, ${\left\{x^{m}\right\}}_{m=1}^{M}$ the Gibbs sampling of one step is given as:

(7) $$\begin{array}{c} \begin{array}{c} \begin{array}{c} v^{\left(0\right)}∼x^{m}\\ h^{\left(0\right)}\sim P\left(h|v^{\left(0\right)}\right) \end{array}\\ v^{\left(1\right)}\sim P\left(v|h^{\left(0\right)}\right) \end{array}\\ h^{\left(1\right)}\sim P\left(h|v^{\left(1\right)}\right) \end{array}$$

Therefore, the update rule of the parameter W can be given by:

(8) $$W\leftarrow W+\varepsilon \left(\langle v_{i}^{\left(0\right)}h_{j}^{\left(0\right)}\rangle -\langle v_{i}^{\left(1\right)}h_{j}^{\left(1\right)}\rangle \right)$$

The parameters c and b, are updated conforming to the same rule. In general, all parameters of each RBM will be continuously optimized until a maximum number of training epochs are reached, which are determined by a human operator. In this way, the iterative training of one RBM is completed and the process will be continued layer by layer until all RBMs in the DBN structure are trained. 

After the DBN is pre-trained, fine-tuning process is utilized in the next step of the DBN training. The fine-tuning process further reduces the training error and improves the classification accuracy of the DBN-based classifier model. As for classification tasks, the output of the DBN calculated from the input sample $x^{m}$ is expected to approximate the label corresponding to $x^{m}$. The BP algorithm is usually utilized to minimize the error between the output of the DBN and the label by adjusting the parameters in the DBN. Supposing that the output of the DBN is $y^{m}$ and the label of $x^{m}$ is $l^{m}$, the training error is defined as:

(9) $$\emptyset_{\mathrm{DBN}}\left(\theta \right)=\frac{1}{M}\overset{M}{\underset{m=1}{\sum }}\left(∥l^{m}-y^{m}{∥}^{2}\right)$$

where θ is the parameter set of the DBN and can be updated as

(10) $$\theta =\theta -\eta \frac{\partial \emptyset_{\mathrm{DBN}}\left(\theta \right)}{\partial \theta }$$

where η is the learning rate. Similarly, this fine-tuning process will be continued until a maximum number of training epochs are reached.

### 2.3. DBN-Based AI Fault Diagnosis Method

**Figure 3:**
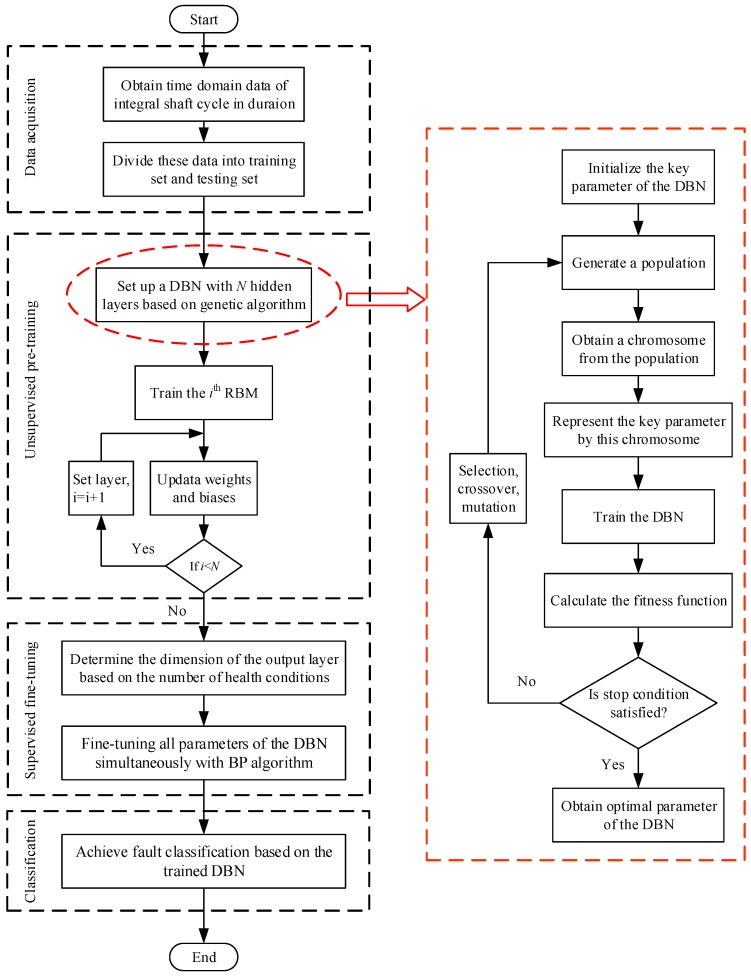
Basic procedures of the proposed deep belief network (DBN) method.

Based on the DBN, this paper proposes a new AI fault diagnosis method that can learn representative features from the raw signals of a gear transmission chain, instead of using features selected by a human operator, to automatically provide accurate classification results. The basic procedures of the proposed method are briefly illustrated in Figure 3.

First, the measured signals must be arranged to fulfil the constraints on the DBN. Conventional AI methods use human-selected features to form the sample set. Here, the measured signals are directly segmented to form the sample sets. The segmentation process has two main steps: (1) determine the axis crossing corresponding to one revolution of the shaft through the measured tachometer signal; and (2) interpolate the time domain data between each of these axis crossings using cubic spline interpolation. The main reason for interpolating the time domain data is that the raw data points between each of these axis crossings may be different because of unstable speed or other complex operating conditions. Each set of time domain data is integrated over the shaft cycle, with durations containing the same number of points to guarantee the capture of all the useful information during each shaft cycle and to correlate the phase features. Following standard practice, the data is interpolated at exactly 2^N^ evenly spaced points, where *N* is the next integer power of two from the length of the raw data. The whole process requires neither conversion between the time and frequency domains nor any other signal processing techniques, thus avoiding some potential problems associated with different signal processing techniques. Finally, these intercepted time domain data form the sample set ${\left\{x^{m},l^{m}\right\}}_{m=1}^{M}$, where $x^{m}$ is the *m*^th^ sample, $l^{m}$ is the classification label of $x^{m}$ and M is the sample size. The number of training and testing samples can be determined for specific applications.

Second, set up a DBN with *N* hidden layers based on genetic algorithm and pre-train layer by layer in an unsupervised manner. It should be noted that the number of RBMs refers to the number of hidden layers because each RBM only contains one hidden layer. Specifically, the input samples are first given to the visible layer of the first RBM. Then the parameters of this RBM are continuously optimized based on the updated rule. While the training epoch reaches its maximum number and the training of the first RBM is accomplished, the hidden layer of this RBM becomes the visible layer of the second RBM. Then the training process is continued for the second RBM. Finally, by training N individual RBMs, all of the hidden layers of the DBN are pre-trained.

Third, determine the dimension of the output layer based on the number of health conditions. Then the BP algorithm is utilized to fine-tune all of the parameters of the DBN by minimizing the error between the output calculated from the input samples and the corresponding labels. Unlike the unsupervised pre-training process, in which each RBM is trained independently, the supervised fine-tuning is applied to all layers simultaneously. Finally, the completely trained DBN is utilized to fault classification of a gear transmission chain.

Besides, it should be noted that some parameters could greatly affect the performance of the DBN. Some researchers have proposed several guidance on how to determine the parameters [43,47]. However, for a specific application, there is no universal rule on the optimized selection of parameter values. In this paper, structural parameters are determined using the genetic algorithm (GA). The main procedures are as follows: (1) Randomly generate an initial population of chromosomes which represent the values of parameters in the DBN. (2) Train the DBN and calculate the fitness function. The fitness function refers to the classification accuracy of the DBN, which is defined as $CA=y_{t}/\left(y_{t}+y_{f}\right)$, where $y_{t}$ and $y_{f}$ represent the number of true and false classifications respectively. (3) Generate a new population in the next generation by the three GA operations, i.e., selection, crossover and mutation, and then continue to search the appropriate parameters. This evolutionary process proceeds until the stop condition is satisfied or a maximum number of generations are reached. 

Through the proposed method, unlabelled time domain data, which are easy to obtain and do not require diagnostic expertise, are utilized and the features are learned from the data instead of being selected by a human operator. Meanwhile, the complex relationship between input data and health conditions can be established to achieve accurate classification. Therefore, the proposed method is capable of the fault diagnosis of a gear transmission chain.

## 3. Fault Diagnosis Based on Supervised Learning Scheme

Signals originating from the gear transmission chain are complicated because of the system’s complexity and its operating conditions [8]. This section describes the rolling bearing experimental system and the problems that limit supervised learning methods applied to the fault diagnosis of rotating machinery.

**Figure 4:**
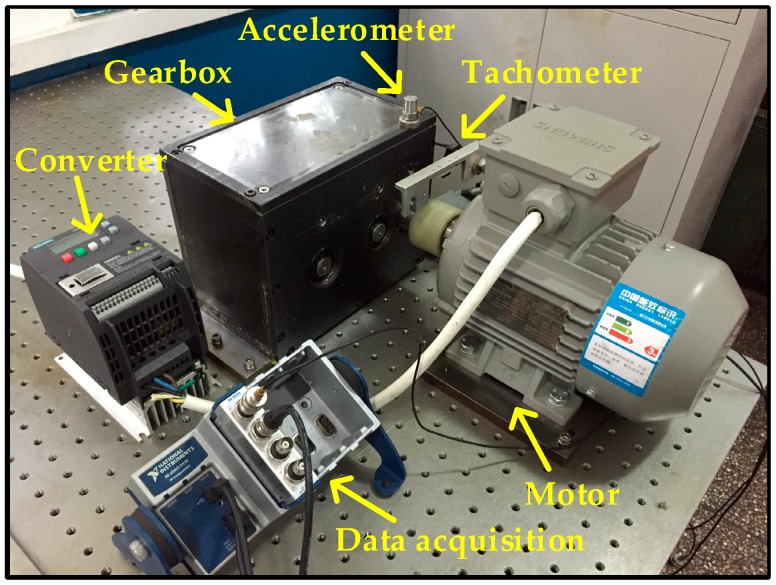
Configuration of the experimental system.

**Figure 5:**
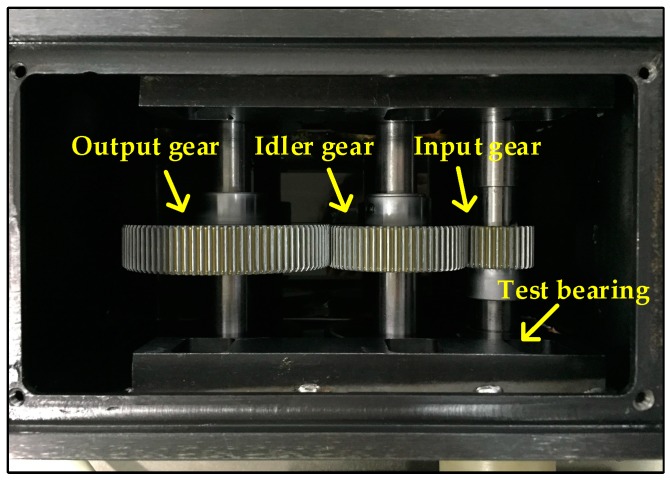
Configuration of the gearbox.

Figure 4 shows the experimental system designed for this work, a two-stage fixed-axis gearbox is driven by a motor (0.75 KW, three-phase, Siemens, Yangzhou, China) and the rotating speed is controlled by the converter (SINAMICS V20, Siemens, Nanjing, China), and a data acquisition system (cDAQ-9234, NI, Austin, TX, USA) is used to collect the data. Figure 5 shows the configuration of the gearbox, there are three shafts inside the gearbox, which are mounted to the gearbox housing with bearings. The input gear has 32 teeth, the idler gear has 64 teeth and the output gear has 96 teeth. In this work, the bearing mounted on the input side of the gearbox is set as test bearing. A mono-axial accelerometer (ICP, KD 1005 L, Yangzhou, China) is mounted on the 12 o’clock position of the input side of the gearbox adjacent to the test bearing for acquiring the vibration signals. A tachometer is used to acquire the input shaft speed in real time. The driven speed of the motor is 2700 rpm, and the sampling frequency is 25.6 kHz.

All bearings used in this work are SKF 6004-2RSH deep groove ball bearing. Some parameters of the bearing are listed as follows: inside diameter: 20 mm; outside diameter: 42 mm; ball diameter: 6.4 mm; pitch diameter: 31 mm; ball number: 9.

**Figure 6:**
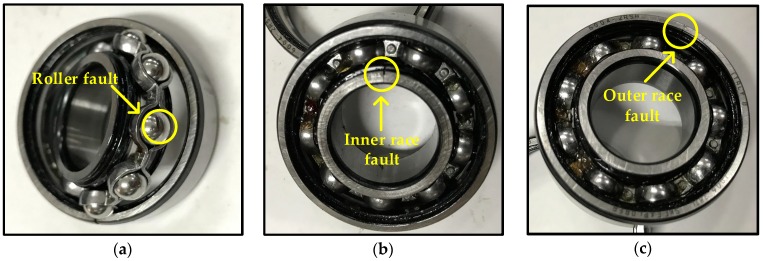
Example of bearing faults: (**a**) roller fault; (**b**) inner race fault; (**c**) outer race fault.

Various faults are introduced to the test bearing using the WEDM method. These faulty bearings are shown in Figure 6. Detailed descriptions of the eight health conditions are summarized in Table 1. Additionally, all the outer race faults are located at 6 o’clock position.

**Figure 7:**
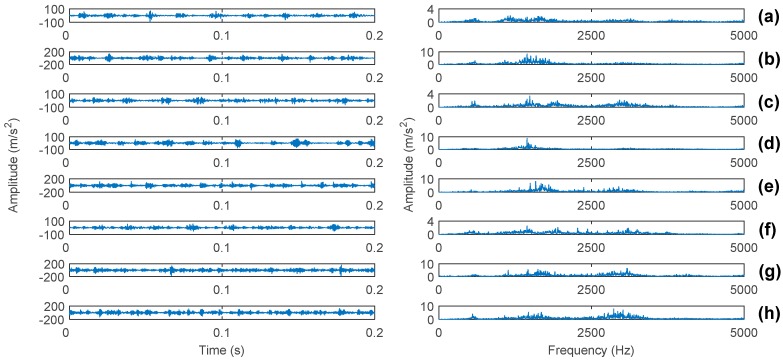
Vibration signals and their corresponding spectrums of the eight health conditions. (**a**–**h**) corresponds to 1–8 states in Table 1.

Figure 7 presents the raw vibration signals of the eight health conditions and their corresponding spectra. However, it is difficult to identify the different health conditions as the characteristic frequencies have no significant difference. Thus, it is necessary to apply a more efficient method to extract the fault characteristics. 

**Figure 8:**
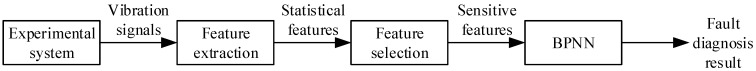
Flowchart of the fault diagnosis method based on supervised learning scheme.

In this work, we utilize the statistical features to characterize the bearing health conditions and employ the BPNN for fault classification. Figure 8 displays the flowchart of fault diagnosis method presented in this section. Here, 10,000 samples can be obtained for each health condition. These samples are randomly partitioned into a training set and a testing set by using *k*-fold cross-validation method, where *k* is chosen as four. Therefore, four subsets are generated where one subset containing 20,000 samples is used as the testing set and the other three subsets containing 60,000 samples are used as the training set. Eleven features in the time domain and four features in the frequency domain, which are listed in Table 2, are calculated from each sample. Specifically, $x_{1}-x_{4}$ may reflect the vibration amplitude and energy in time domain. $x_{5}-x_{11}$ may represent the time series distribution of the signal in time domain. $x_{12}$ may indicate the vibration energy in frequency domain. $x_{13}-x_{14}$ may reflect the position change of the main frequencies. $x_{15}$ may describe the convergence of the spectrum power. As the amplitude and distribution of the vibration signals may change when faults occur, these changes can be captured by the listed statistical features in Table 2.

**Figure 9:**
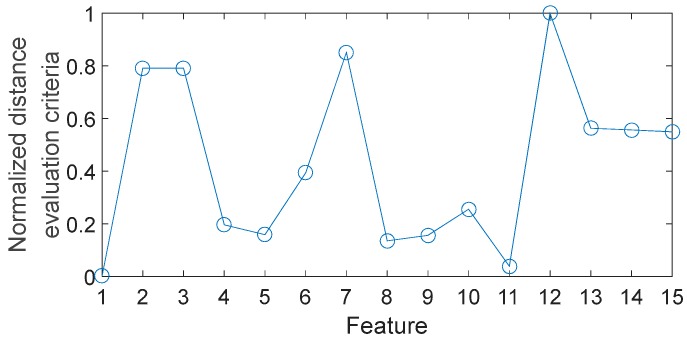
Normalized distance evaluation criteria of 15 features.

**Figure 10:**
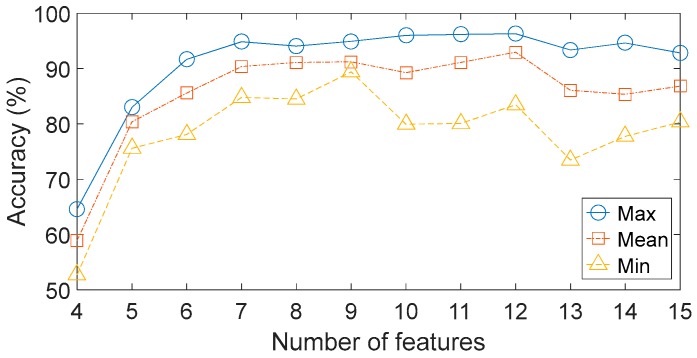
Performances of different feature numbers.

To reduce feature dimensionality and improve classification accuracy, feature selection is critical to the subsequent classification. Several researchers have proposed effective methods of feature selection. Because of its simplicity and reliability, the distance evaluation technique (DET) is widely used to feature selection [48]. This method has three main steps: (1) calculate the average distance of each feature inside the same condition pattern $S_{j}^{\left(w\right)}$, where j is the feature number of each sample; (2) calculate the average distance of each feature between different condition pattern $S_{j}^{\left(b\right)}$; (3) define the distance evaluation criteria as $\alpha_{j}=S_{j}^{\left(b\right)}/S_{j}^{\left(w\right)}$. Following standard practice, we can obtain the normalized distance evaluation criteria ${\bar{\alpha }}_{j}=\alpha_{j}/\max\left(\alpha_{j}\right)$. It is clear that the lager ${\bar{\alpha }}_{j}$ means that the corresponding feature is better to increase the separation among different conditions. In this work, the DET is applied to evaluate the importance of all features. The normalized distance evaluation criteria of 15 features are shown in Figure 9. The next problem is how many features should be selected. It is obvious that fewer features may lead to lack of critical information, while a larger number of features do not necessarily result in higher classification accuracy because there may be irrelevant or redundant information in these features. Here, different numbers of features with lager criteria are evaluated by the BPNN-based method respectively. The designed BPNN has three hidden layers: the unit numbers of the first layer, the second layer and the third layer are 100, 50 and 10 respectively. The maximum training epoch is 500, and the learning rate is 0.05. The results are shown in Figure 10. The classification accuracy defined in this paper refers to the ratio of samples that are correctly classified to the total sample set, which is defined as follows:

(11) $$CA=\frac{y_{t}}{y_{t}+y_{f}}$$

where $y_{t}$ and $y_{f}$ represent the number of true and false classifications respectively. 

The results show the poor accuracies when only four or five features are used. Meanwhile, when the number of features is larger than five, the average accuracies improve slightly but are still unsatisfactory under several engineering applications. The performances of different feature subsets have not improved when increasing the number of features, which implies that the original feature set contains irrelevant or redundant information. In addition, the performances for each feature subset vary greatly over the four trials. For instance, when seven features are included in the feature subset, the highest and the lowest accuracies are respectively to be 94.86% and 84.79%. These results indicate that the BPNN-based method offers poor stability and unacceptable robustness.

**Figure 11:**
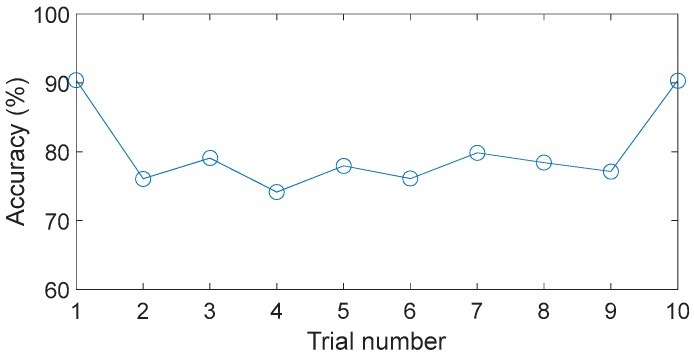
Performances of different feature types.

The performances of different feature types are also evaluated in this work. Ten feature subsets, each containing seven features, are selected from the original feature set, and the features in each subset are not necessarily the same. The features in the first subset are selected based on DET, whereas the others are selected randomly. The results of this analysis are shown in Figure 11, where it is clear that the performances of the ten feature subsets vary greatly, implying that some typical features are inappropriate for fault diagnosis in this case. Besides, several feature subsets with the number of features respectively to be 1~15 have been investigated and the results of these cases have no significant difference.

From the above analysis, it is clear that learning representative features for each task is crucial for classification. In this work, 15 statistical features in the time domain and frequency domain are extracted from the raw signals and the DET is used to select the sensitive features. It is feasible that by employing other appropriate data analysis algorithms, better classification accuracy may be obtained. However, this process largely depends on prior knowledge of signal processing techniques and diagnostic expertise. In addition, the signal processing techniques adopted to solve one specific issue may not be suitable for others. Because of these problems, unsupervised feature learning is expected to be more effective against the challenges facing fault diagnosis of gear transmission chains.

## 4. Fault Diagnosis Based on the Proposed Method

Rolling bearings and gears are the key components in rotating machinery. Faults occurring in these components must be detected as early as possible to avoid fatal breakdowns of machines. In this section, two experiments of rolling bearings and gearbox are used to validate the proposed method.

### 4.1. Fault Diagnosis of Rolling Bearing in Gear Transmission Chain

In this subsection, the fault signals collected from the experimental system described in Section 3 are used to validate the performance of the proposed method. In this study, 10,000 samples can be obtained for each health condition. These samples are randomly partitioned into a training set and a testing set by using *k*-fold cross-validation method, where *k* is chosen as four. Therefore, four subsets are generated where one subset containing 20,000 samples is used as the testing set and the other three subsets containing 60,000 samples are used as the training set. Each sample contains the time domain data from duration of three shaft cycles, with 2048 data points. The DBN has 2048 input units, equal to the dimension of the samples, and eight output units, equal to the number of health conditions. There is one hidden layer with 1000 hidden units, which is determined through the genetic algorithm. In the process of unsupervised pre-training, the maximum training epoch is 25, the learning rate is 0.005, the momentum is 0.5 and the biases are initialized to zero. In the process of supervised fine-tuning, the weights are initialized randomly and the maximum training epoch is 10.

For comparison, the BPNN and SVM are trained using the same data for fault diagnosis as well. The BPNN has the same architecture as the DBN and is trained by the same parameters. The SVM uses RBF kernel function as the basic function and the kernel width is set to be two. The classification results are shown in Table 3. The results clearly show that with the proposed method, the average accuracy is 99.26% with a standard deviation of 0.02%, which means that the proposed method can distinguish the eight health conditions of rolling bearing with a high accuracy. In contrast, the classification accuracies using the BPNN-based method and the SVM-based method are comparatively poor. The average accuracies of these two methods are 82.23% and 94.50% respectively, confirming the superiority of the proposed method to the conventional AI methods. To analyze the classification results of each health condition more thoroughly, the confusion matrix of one trial which produced by the proposed method is presented in Table 4. It shows that both the producer’s accuracies and the user’s accuracies of each health condition retain at high level with strong robustness, confirming that the proposed method can effectively and stably distinguish not only bearing fault categories but also fault severities.

**Figure 12:**
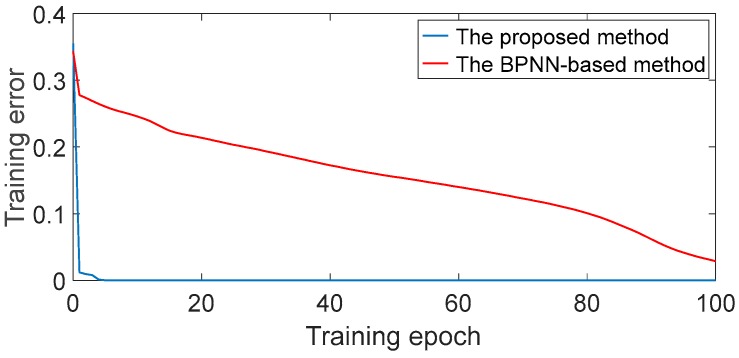
Curves of the training error of the proposed method and the BPNN-based method.

Besides, the training errors (MSE) of DBN and BPNN in one trial are shown in Figure 12. It is noticed that the training error converges rapidly to nearly zero within 10 epochs when using the proposed method. Accordingly, it is feasible to achieve accurate fault classification when the training epoch is set to 10 in this experiment. By contrast, the training error converges slowly when using the BPNN-based method, leading to the unsatisfied classification accuracy. This result indicates that the proposed method indeed has the capacity to model complex rolling bearing data accurately and is more robust than the BPNN-based method. In addition, it should be note that the DBN needs more training time than conventional artificial neural network methods because of the unsupervised pre-training and the deep architecture. However, it has been proved that the parallel processing technique is conductive to improve the computation speed. Some researchers have pointed out that the fast graphics processing units (GPUs) can greatly reduce the time of training networks [27,39]. Therefore, with the development of hardware and data processing technology, we can train the DBN faster.

As mentioned above, in the proposed method, the unsupervised process aims to adaptively exploit representative features characterizing the health conditions of machinery from the unlabelled time domain data instead of relying on diagnosticians. Besides, when designing the architecture of the DBN, in general the unit number of the next layer is smaller than that of the previous layer so that the data can be compressed layer by layer. Therefore, the unsupervised process in the DBN can correspond to the feature extraction and feature selection in the conventional AI method. These features are considered as the input of the output layer of the DBN. The output layer of the DBN can be treated as a classifier to achieve fault classification after supervised fine-tuning. In this case, the DBN has one hidden layer with 1000 hidden units. Therefore, 1000 features are exploited from each sample through unsupervised feature learning and then are used to fault classification. To verify the ability of these 1000 features in characterizing the health conditions of rolling bearing, we set these features as the input of two well-known classifiers, i.e., BPNN and SVM, to achieve fault classification. The average accuracy using the unsupervised feature learning and BPNN is 98.94%, with a standard deviation of 0.04%. The average accuracy using the unsupervised feature learning and SVM is 98.49%, with a standard deviation of 0.11%. Compared to the results listed in Table 3, the accuracies improve significantly when using unsupervised feature learning, which illustrates that the proposed method could adaptively exploit the representative features characterizing the health conditions of rolling bearing. In addition, in the proposed method, after the DBN is pre-trained, the fine-tuning process further reduces the training error and improves the classification accuracy of the DBN-based classifier. Therefore, the performance of the proposed method is superior to the conventional AI methods.

**Figure 13:**
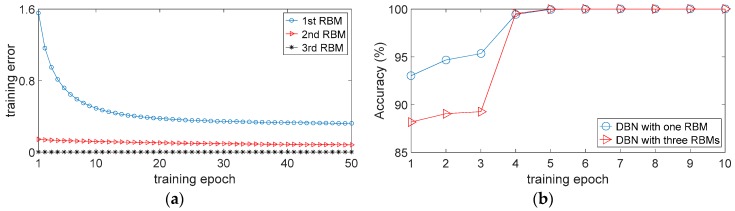
The performance of two different DBN architectures: (**a**) training errors of each RBM; (**b**) training accuracies of two different DBN architectures.

The architecture selection is an important process for most neural network models. Certain architecture parameters, such as the number of hidden layers and the number of hidden units per layer, are critical to the performance of the neural network model [47]. In the proposed method, the DBN contains one hidden layer with 1000 hidden units, which is determined through the genetic algorithm. In a previous study [43], the authors presented a simple idea to decide the architecture: the unit number of the next layer is smaller than that of the previous layer so that the feature learning process can be viewed as a data compression process. Based on this idea, we also set up a DBN with three hidden layers for comparison. The unit number of the first hidden layer, the second hidden layer and the third hidden layer are 1000, 500 and 200 respectively. Figure 13a shows the training errors of each RBM. It is noticed that the training error declines rapidly within the first 20 epochs when training the first RBM. However, the training error no longer declines almost after 25 epochs, which means that the optimization of the parameters such as weights and biases seems to reach a stage of stagnation. A similar situation has occurred when training the second and third RBM, the training error shows no distinct decline within the 50 epochs. Figure 13b shows the training accuracies of two different DBN architectures. It is noticed that the training accuracies of both DBN architectures reach almost 100% within five epochs. Nevertheless, an interesting phenomenon is also observed that in the first three epochs the performance of the DBN with three RBMs is inferior to that of the DBN with one RBM. This phenomenon shows that the DBN with one RBM is capable enough to adaptively exploit representative features by unsupervised pre-training. Although the second and third RBMs further compress data, they may also lead to reconstruction errors as well as higher computational cost. Therefore, a DBN contains one hidden layer with 1000 hidden units is applied here for the classification task.

**Figure 14:**
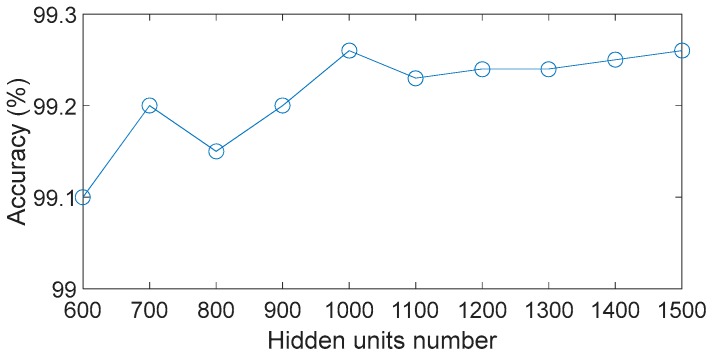
Diagnosis results for different numbers of hidden units.

To further evaluate the performance of the proposed method, we change the number of hidden units, and the results are shown in Figure 14. In this analysis, all the classification accuracies are over 99% regardless of the number of hidden units, which means that the DBN has the ability to self-regulate and effectively learn the complex non-linear characteristics of health conditions even if some parameters have not been fully optimized. Also of note, the genetic algorithm is an approximate optimization algorithm, therefore the DBN containing one hidden layer with 1000 hidden units does not necessarily produce the highest classification accuracy. Nonetheless, to a certain extent, the advantages of the DBN reduce the dependence on the optimization algorithm.

Moreover, Table 5 shows the influence of sample size on the performance of the proposed method. As a total of 80,000 samples can be obtained, four combinations between the training samples and the testing samples are tested, i.e., 40,000 training samples & 40,000 testing samples, 50,000 training samples & 30,000 testing samples, 60,000 training samples & 20,000 testing samples and 70,000 training samples & 10,000 testing samples. 

It can be seen from Table 5 that the accuracies of all trials are higher than 98%, confirming the strong robustness of the proposed method to different choices of training & testing samples. Besides, it shows that more training samples tends to show better performance compared with few training samples. The main reason is that more training samples contain sufficient and complementary information related to the health conditions of machinery so that the DBN can exploit representative features in the feature space, while few samples may lead to an estimation deviation from the feature space. 

### 4.2. Fault Diagnosis of Gear Transmission Chain

**Figure 15:**
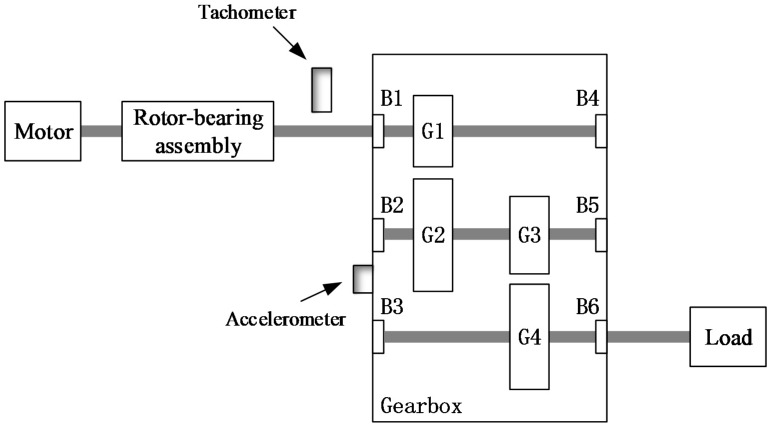
Layout of the two-stage gearbox system used to validate the performance of the proposed method.

The two-stage gearbox vibration data used in this subsection are provided by the 2009 Prognostics and Health Management Data Challenge [49,50,51]. Figure 15 displays a diagram of the experimental system used to collect the data, which contains a rotor-bearing assembly, a two-stage fixed-axis gearbox, a motor for driving, and a magnetic brake for loading. There are three shafts inside the gearbox, which are mounted to the gearbox housing with six rolling bearings. **#**Gear 1 has 32 teeth, **#**Gear 2 has 96 teeth, **#**Gear 3 has 48 teeth, and **#**Gear 4 has 80 teeth. 

Eight experiments are conducted under different health conditions. These conditions involve multiple fault categories for the gear, bearing, and shaft components, as described in Table 6. Some components that have not been listed in Table 7, including **#**Gear 2, **#**Bearing 4, **#**Bearing 5, and **#**Bearing 6, are in health condition. In comparison with the experiment in the previous section, more fault categories and different fault locations in the gearbox are investigated in this experiment. An accelerometer mounted on the input side of gearbox is used to acquire vibration signals, and a tachometer mounted on the input shaft is used to acquire the real-time input shaft speed. The data are collected under the no-load and the maximum load (10 in-lbs.) conditions with a sampling frequency of 66.67 kHz. The motor speed is 3000 rpm. 

In this study, 220 samples can be obtained for each health condition. These samples are randomly partitioned into a training set and a testing set by using *k*-fold cross-validation method, where *k* is chosen as four. Therefore, four subsets are generated where one subset containing 440 samples is used as the testing set and three subsets containing 1320 samples are used as the training set. Each sample contains the time domain data from duration of three shaft cycles, with 4096 data points. The data collected under different loads are not separated, so that the same health condition under different loads is treated as one class.

Similarly, three methods are used to process the dataset and the classification results are shown in Table 7. It is apparent that the performance of the BPNN-based method and the SVM-based method are inferior to that of the proposed method. For the proposed method, the average accuracy is 100%, which means all the samples are correctly classified. However, for the BPNN-based method, the average accuracy is 80.97%, with a standard deviation of 5.14%. For the SVM-based method, the average accuracy is 91.82%, with a standard deviation of 2.55%. This illustrates the superiority of the proposed method to the other two methods in terms of distinguishing the various fault categories and different fault locations of the gearbox. 

In this experiment, as the hidden layer of the DBN has 1000 hidden units, 1000 features are exploited from each sample through unsupervised feature learning and then are used to fault classification. Similarly, we set these features as the input of BPNN and SVM to achieve fault classification, and the average accuracies are 100% when using both methods. Compared to the results listed in Table 7, the accuracies improve significantly when using unsupervised feature learning. These results illustrate that the proposed method can adaptively exploit the fault features of gearbox.

## 5. Discussion

The analysis in Section 4 shows that the proposed method can effectively learn relevant features and accurately classify various health conditions in rotating machinery. The experiment in Section 4.1 is aimed at the fault diagnosis of rolling bearings in the gearbox. The collected data are very complicated because of the system’s structure and operating conditions. Nevertheless, the average accuracy of the proposed method is 99.26%, with a standard deviation of 0.02% when the combination of sample size is 60,000 training samples & 20,000 testing samples, indicating that this method can effectively and stably distinguish not only bearing fault categories but also fault severities. The experiment in Section 4.2 investigates the fault diagnosis of a gearbox, including more fault categories and different fault locations in the gearbox. The performance of the proposed method remains excellent. In contrast, the performance of the BPNN-based method and the SVM-based method in both analyses are comparatively poor. These results confirm that the unsupervised feature learning model with deep architectures has superior capacity to accurately model complexly structured data compared to the shallow neural network model. Besides, many researches focus on the application of deep learning in the fault diagnosis of rotating machinery. In a previous study [39], a deep statistical feature learning for vibration measurement has been proposed to diagnose fault patterns in rotating machinery. Two typical rotating machinery systems (gearbox fault diagnosis system and bearing fault diagnosis system) are constructed to validate its method. In the first experiment, the method is used to distinguish ten gear health conditions under different loads and 95.17% classification accuracy is obtained. In the second experiment, the method is used to distinguish seven bearing faults and 91.75% classification accuracy is obtained. Both the design of experiments and fault categories are similar to that in this paper, and the results show that the proposed method can obtain higher classification accuracies compared with the deep learning method in [39].

Feature extraction and feature selection are crucial steps in fault diagnosis because the relevance of the extracted features directly affects the classification accuracy. In this work, we present the results of the BPNN-based method applied to raw data and different time domain features, and it is found that the classification accuracies vary dramatically at unsatisfactory levels. To overcome these limitations, developing robust features that capture the relevant information for each issue may improve the classification accuracy. However, this process is time-consuming and largely depends on diagnostic expertise. In contrast, the proposed method uses unsupervised feature learning to obtain representative features from the raw data and achieve accurate classification. This offers the advantage of using raw data instead of extracting features based on the expertise of a human operator. The whole process requires neither conversion between the time and frequency domains nor other signal processing techniques, thus avoiding some potential problems associated with different signal processing techniques. To verify the ability of the proposed method in adaptively exploiting fault features, we set the features exploited through unsupervised feature learning as the input of BPNN and SVM to achieve fault classification. In the first experiment, the average accuracy is 98.94% when using the unsupervised feature learning and BPNN, and 98.49% when using the unsupervised feature learning and SVM. In the second experiment, the average accuracies even reach 100%. Compared to the results list in Table 3 and Table 7, the accuracies are much higher than that of the conventional BPNN-based method and SVM-based method. The results reveal that the proposed method could adaptively exploit the fault features of balling bearings and gearbox.

The architecture selection is an important process for most neural network models. In this paper, the DBN contains one hidden layer with 1000 hidden units, which is determined through the genetic algorithm. To further evaluate the proposed method, we changed the number of hidden units and calculated the classification accuracy. This analysis showed that the limited variation of some architecture parameters has little effect on the performance of the DBN.

The influence caused by different sample sizes has also been considered. In Section 4.1, four combinations between the training samples and the testing samples are tested to evaluate the robustness of the proposed method. The results show that the accuracies of all trials are higher than 98%, confirming the strong robustness of the proposed approach to different choices of training & testing samples.

In this paper, the unlabelled time domain data of integrated shaft cycles are utilized for classification. The only pre-processing step is to apply the tachometer signal to determine the shaft cycle duration. Beyond that, no other signal processing technique is required. Currently, some data acquisition systems can adjust the data sample rate based on a tachometer signal so that 2^N^ data points are captured per cycle of rotation. In this way, we can directly train the DBN using raw data in the time domain without any pre-processing.

## 6. Conclusions

This paper proposes a DBN-based AI method for the fault diagnosis of a gear transmission chain. In this method, the DBN based classifier is first pre-trained layer by layer in an unsupervised manner and then fine-tuned with a BP algorithm under supervision. Besides, the genetic algorithm is used to optimize the structural parameters of the network. In contrast to the supervised neural network, the proposed method takes full advantage of unsupervised feature learning to extract features from the unlabelled time domain data instead of relying on a human operator to extract features. Therefore, the proposed method depends less on field expertise or prior knowledge of diagnostic techniques. Moreover, the proposed method has superiorities to model complex structured data, thus can discover the discriminative information of these data and achieve accurate classification.

Two experiments, i.e., rolling bearing faults and gearbox faults, are conducted to verify the performances of the proposed method. Various fault categories, fault locations and fault severities under different loading conditions are considered in the experiments. The fault classification accuracies are 99.26% for rolling bearings and 100% for gearbox when using the proposed method. Besides, the confusion matrix shows that the accuracy of each health condition retains at high level with strong robustness. In contrast, the performance of the BPNN-based method and the SVM-based method in both experiments are comparatively poor. The above results show that the proposed method performs better in the classification of gear transmission chain faults compared to the conventional AI methods. Besides, the vibration data utilized here is obtained from only one sensor, our future work will focus on a more comprehensive fault diagnosis of rotating machinery based on the multi-sensor data fusion and deep learning methods.

## Figures and Tables

**Table 1:** Description of bearing faults.

Label	Fault Description	Fault Width (mm)	Fault Depth (mm)
1	Normal	0	0
2	Inner race serious fault	0.8	0.2
3	Outer race serious fault	0.8	0.2
4	Roller fault	0.5	0.2
5	Inner race minor fault	0.3	0.2
6	Outer race minor fault	0.3	0.2
7	Hybrid serious fault (inner race serious fault, outer race serious fault and roller fault)	0.8/0.8/0.5	0.2/0.2/0.2
8	Hybrid minor fault (Inner race minor fault, outer race minor fault and roller fault)	0.3/0.3/0.5	0.2/0.2/0.2

**Table 2:** The statistical features in the time domain and frequency domain.

Feature	Equation	Feature	Equation
Mean	$x_{1}=\frac{1}{N}\sum_{i=1}^{N}x_{i}$	Rectified mean	$x_{2}=\frac{1}{N}\sum_{i=1}^{N}|x_{i}|$
Peak to peak value	$x_{3}=\max\left(x_{i}\right)-\min\left(x_{i}\right)$	Root mean square	$x_{4}=\sqrt{\frac{1}{N}\sum_{i=1}^{N}{x_{i}}^{2}}$
Standard deviation	$x_{5}=\sqrt{\frac{\sum_{i=1}^{N}{\left(x_{i}-x_{1}\right)}^{2}}{N-1}}$	Skewness	$x_{6}=\frac{\sum_{i=1}^{N}{\left(x_{i}-x_{1}\right)}^{3}}{\left(N-1\right){x_{5}}^{3}}$
Kurtosis	$x_{7}=\frac{\sum_{i=1}^{N}{\left(x_{i}-x_{1}\right)}^{4}}{\left(N-1\right){x_{5}}^{4}}$	Impulse factor	$x_{8}=\frac{\max\left(x_{i}\right)}{x_{2}}$
Shape factor	$x_{9}=\frac{x_{4}}{x_{2}}$	Crest factor	$x_{10}=\frac{\max\left(x_{i}\right)}{x_{4}}$
Coefficient of variation	$x_{11}=\frac{x_{5}}{x_{1}}$	Mean frequency	$x_{12}=\frac{\sum_{k=1}^{K}s\left(k\right)}{K}$
Frequency center	$x_{13}=\frac{\sum_{k=1}^{K}f_{k}s\left(k\right)}{\sum_{k=1}^{K}s\left(k\right)}$	Root mean square frequency	$x_{14}=\sqrt{\frac{\sum_{k=1}^{K}{f_{k}}^{2}s\left(k\right)}{\sum_{k=1}^{K}s\left(k\right)}}$
Standard deviation frequency	$x_{15}=\sqrt{\frac{\sum_{k=1}^{K}{\left(f_{k}-x_{fc}\right)}^{2}s\left(k\right)}{\sum_{k=1}^{K}s\left(k\right)}}$		

Note: $x_{i}$ is the *i*^th^ value of signal x, N is the number of data points. s(k) is a spectrum for k=1,2,⋯,K, K is the number of spectrum lines; $f_{k}$ is the frequency value of the *k*^th^ spectrum line.

**Table 3:** Classification results of rolling bearing.

Methods	Average Accuracy (%)	Standard Deviation (%)
The proposed method	99.26	0.02
The BPNN-based method	82.23	5.76
The SVM-based method	94.50	0.18

**Table 4:** The confusion matrix produced by the proposed method.

Label		Predicted								Sum	PA (%)
		1	2	3	4	5	6	7	8		
**Actual**	**1**	2470	1	1	0	6	1	21	6	2506	98.56
	**2**	1	2454	0	1	0	7	0	0	2463	99.63
	**3**	0	0	2501	0	1	0	3	7	2512	99.56
	**4**	0	3	0	2509	0	0	0	0	2512	99.88
	**5**	0	0	0	0	2545	0	0	0	2545	100
	**6**	1	13	0	1	2	2438	6	2	2463	98.99
	**7**	3	1	1	1	1	0	2507	11	2525	99.29
	**8**	9	4	3	0	4	0	28	2426	2474	98.06
	**Sum**	2484	2476	2506	2512	2559	2446	2565	2452	20,000	
**UA (%)**		99.44	99.11	99.80	99.88	99.45	99.67	97.74	98.94		

Overall accuracy: 99.25%; Kappa coefficient: 0.9914. Note: PA means the Producer’s Accuracy, UA means the User’s Accuracy.

**Table 5:** The influence of sample size on the performance of the proposed method.

Number of Training Samples & Testing Samples	Average Accuracy (%)	Standard Deviation (%)
40,000 & 40,000	98.76	0.05
50,000 & 30,000	99.08	0.05
60,000 & 20,000	99.26	0.02
70,000 & 10,000	99.32	0.08

**Table 6:** Experimental conditions for the gear transmission chain analysis.

#	Gear			Bearing			Shaft	
	G1	G3	G4	B1	B2	B3	Input	Output
1	√	√	√	√	√	√	√	√
2	Chipped	Eccentric	√	√	√	√	√	√
3	√	Eccentric	√	√	√	√	√	√
4	√	Eccentric	Broken	Ball	√	√	√	√
5	Chipped	Eccentric	Broken	Inner	Ball	Outer	√	√
6	√	√	Broken	Inner	Ball	Outer	Imbalance	√
7	√	√	√	Inner	√	√	√	Keyway sheared
8	√	√	√	√	Ball	Outer	Imbalance	√

**Table 7:** Classification results of gear transmission chain.

Methods	Average Accuracy (%)	Standard Deviation (%)
The proposed method	100	0
The BPNN-based method	80.97	5.14
The SVM-based method	91.82	2.55
